# Electrospray Quadrupole Travelling Wave Ion Mobility Time-of-Flight Mass Spectrometry for the Detection of Plasma Metabolome Changes Caused by Xanthohumol in Obese Zucker (fa/fa) Rats

**DOI:** 10.3390/metabo3030701

**Published:** 2013-08-13

**Authors:** Samanthi I. Wickramasekara, Fereshteh Zandkarimi, Jeff Morré, Jay Kirkwood, LeeCole Legette, Yuan Jiang, Adrian F. Gombart, Jan F. Stevens, Claudia S. Maier

**Affiliations:** 1 Department of Chemistry, Oregon State University, Corvallis, OR 97331, USA; E-Mails: samanthi.wickramasekara@oregonstate.edu (S.I.W.); zandkarf@onid.orst.edu (F.Z.); jeff.morre@oregonstate.edu (J.M.); 2 Department of Pharmaceutical Sciences, Oregon State University, Corvallis, OR 97331, USA; E-Mails: kirkwooj@onid.orst.edu (J.K.); legettel@onid.orst.edu (L.L.); fred.stevens@oregonstate.edu (J.F.S.); 3 Linus Pauling Institute, Oregon State University, Corvallis, OR 97331, USA; E-Mail: adrian.gombart@oregonstate.edu; 4 Department of Statistics, Oregon State University, Corvallis, OR 97331, USA; E-Mail: yuan.jiang@stat.oregonstate.edu; 5 Department of Biochemistry and Biophysics, Oregon State University, Corvallis, OR 97331, USA

**Keywords:** obesity, xanthohumol, travelling wave ion mobility, mass spectrometry, lipidomics

## Abstract

This study reports on the use of traveling wave ion mobility quadrupole time-of-flight (ToF) mass spectrometry for plasma metabolomics. Plasma metabolite profiles of obese Zucker fa/fa rats were obtained after the administration of different oral doses of Xanthohumol; a hop-derived dietary supplement. Liquid chromatography coupled data independent tandem mass spectrometry (LC-MS^E^) and LC-ion mobility spectrometry (IMS)-MS^E^ acquisitions were conducted in both positive and negative modes using a Synapt G2 High Definition Mass Spectrometry (HDMS) instrument. This method provides identification of metabolite classes in rat plasma using parallel alternating low energy and high energy collision spectral acquisition modes. Data sets were analyzed using pattern recognition methods. Statistically significant (*p* < 0.05 and fold change (FC) threshold > 1.5) features were selected to identify the up-/down-regulated metabolite classes. Ion mobility data visualized using drift scope software provided a graphical read-out of differences in metabolite classes.

## 1. Introduction

Metabolomics, the analysis of endogenous metabolites (<1,500 Dalton) in complex mixtures, is heavily based on the combination of separation techniques and mass spectrometry. The predominant analytical methods are hyphenated approaches combining gas chromatography with mass spectrometry (GC-MS) [1,2], liquid chromatography and mass spectrometry (LC-MS) [3,4] and capillary electrophoresis and mass spectrometry (CE-MS) [5,6,7]. As an analytical science, advances in metabolomics are predominately achieved by technology advancements [8,9]. During the last decade, mass analyzers have seen significant improvements in resolving power, mass accuracy and sensitivity. The combination of soft ionization techniques, such as atmospheric pressure chemical ionization (APCI) and electrospray ionization (ESI), and hyphenated LC-MS/MS techniques are the current method of choice for the analysis of polar metabolites [10,11].

The precise and accurate quantification of metabolites is the key objective of targeted metabolomics. Targeted metabolite profiling analyses are often conducted using triple quadrupole instruments operated in the selected reaction monitoring mode. Q-trap-type instruments allow data-dependent acquisition modes that combine selected reaction monitoring and product ion scanning modes, providing good quantification of analytes and increased confidence in metabolite identification [4,12,13]. In contrast, the main objective of untargeted metabolomics is the detection and identification of as many metabolites as possible. Accurate mass measurement of the metabolites is of paramount importance for the success of untargeted metabolome analyses, because the mass-to-charge ratio (*m/z*) value of the metabolite ion is used initially for searching metabolite databases. Current mass analyzers that enable accurate mass measurements with high resolution are modern time-of-flight (ToF) instruments, Fourier transform (FT) orbitraps and ion cyclotron resonance (ICR) analyzers. However, if metabolite identification is the goal of the analysis, the mass information needs to be complemented with structural data that can be obtained by performing collision-induced dissociation (CID). The most common acquisition mode in untargeted metabolomic experiments is the acquisition of full scan data and product ion data using data-dependent acquisition (DDA) modes [14,15]. DDA acquisitions are technically realized in hybrid tandem mass spectrometry instruments by combining multiple mass analyzer, such as the Q-ToF-type instruments, quadrupole orbitraps (Q Exactive), and combinations of linear trap quadrupole (LTQ) instruments with high resolution mass analyzer, e.g. LTQ-orbitraps and the LTQ-FT instruments. A possible drawback of DDAs is that a substantial proportion of the acquisition cycle is spent acquiring product ion spectra; therefore, the accuracy with which the intensities of the precursor ions are measured in the first MS stage (MS1) are compromised [16]. More recently, data-independent acquisition (DIA) methods have become available that do not select specific precursor ions for gas phase fragmentation and acquire ion signals for precursor and fragment ion seemingly uninterrupted. It is for this reason that DDA methods deliver more accurate quantitative precursor ion signals suited for quantitative studies [17,18]. Independent of the mode of acquisition, untargeted metabolomic experiments greatly benefit from the availability of high resolution accurate mass measurements of precursor and product ions, enabling the assignments of metabolites with greater confidence.

Recent developments in quadrupole time-of-flight (q-ToF) hybrid instruments led to improvements in sensitivity, duty cycle and dynamic range. In addition, the introduction of collision energy profiles for the non-selective MS/MS acquisition mode, so-called MS^E^, provides an untargeted and unbiased methodology for metabolite fragmentation [17,19,20]. The combination of MS^E^ acquisitions with the separation power of Ultra Performance Liquid Chromatography (UPLC) enables conducting many precursor and neutral loss acquisitions over a single experimental run and can overcome duty cycle issues associated with similar types of experiments involving other scanning-type instruments [21,22]. Furthermore, MS^E^ supports the analysis of any compound of interest irrespective of its signal intensity and has been applied for many proteomic and metabolomic studies [17,23,24,25]. Additionally, MS^E^ in conjunction with a high-end ToF analyzer provides exact mass information for precursor and fragment ions, a prerequisite for determining elemental compositions and minimizing false positive identifications.

The combination of ion mobility spectrometry (IMS) with hybrid mass spectrometry (MS) instruments offers an additional dimension of separation for complex mixtures. In ion mobility spectrometry, ions are separated as they drift through gas (He, Ar, CO_2_) under the influence of an electric field. The rates of drift depend on the size, charge and the collisional cross section of the respective ions. Adding the drift time information may add an additional level of specificity to the mass-to-charge (*m/z*) analysis. The integration of ion mobility spectrometry with mass spectrometry (IMS-MS) provides a rapid method for the analysis of isobaric molecules, such as small molecule regioisomers and polypeptides adopting different conformer structures [26,27,28]. In the current report, a commercial ion mobility mass spectrometry instrument that features a Triwave^TM^ geometry in which the ion mobility cell (IMS T-wave) is placed between two traveling wave ion guides (trap T-wave and transfer T-wave) was used. These devices can be used as either ion guides or collision cells, depending on the collision energy voltages applied. Fragmentation of ions can be induced in both devices, the trap (pre-IMS separation) and/or the transfer (post-IMS separation) [29]. With the trap-only fragmentation, the ions will undergo high energy collisions before they enter into the IMS T-wave region. Then, the fragments will be separated in the second travelling wave device based on their collisional cross sections and be guided to the ToF detector with minimum or no collision at the transfer T-wave cell. With the transfer-only fragmentation, the ions pass through the first travelling wave cell with no collision energy applied, and the parent ions will separate by the IMS T-wave cell, followed by fragmentation in the transfer T-wave region. When the ions fragment in this region, they retain the original drift time of the parent ion., The combined use of both retention time- and drift time-alignment of parent and fragment ions enables the identification of metabolites with high confidence even in complex matrices. Another way to get more structural information on the ions is to use time-aligned parallel (TAP) fragmentation, in which both trap T-wave and transfer T-wave cells are kept at high voltage. In the TAP mode, the ion of interest will be selected in the quadrupole region and will undergo first generation fragmentation in the trap cell, and the resulting fragment ions will separate in the ion mobility cell. The mobility-separated fragment ions will undergo further fragmentation, resulting in second generation fragment ions, which are time-aligned with the first generation fragment ions [26]. In this study, we used ion mobility separation in combination with MS^E^ acquisition for the identification of plasma metabolites from obese Zucker rats. In this method, precursor ions are separated by ion mobility and, then, subjected to MS^E^ acquisition, which applies parallel low and high energy cycles for achieving exact mass analyses and fragment ion generation. In this mode, the fragment ions are aligned with their precursors with respect to their drift times.

Xanthohumol (XN), a prenyl flavonoid extracted from the hop plant, has gained much attention, due to its anti-inflammatory, antimicrobial, antioxidant and anticancer properties [30,31]. Work by Legette *et al.* indicated that the XN lowers body weight and improved glucose homeostasis in obese Zucker (fa/fa) rats [32]. As part of a larger ongoing effort to investigate the possible health promoting effects of XN in obesity, the objective of this study was to apply electrospray travelling wave ion mobility mass spectrometry (ESI-T-wave-IMS-MS) to investigate the plasma metabolome of obese Zucker (fa/fa) rats treated with different oral doses of xanthohumol.

## 2. Results and Discussion

### 2.1. HPLC-TOF-MS^E^ Analysis

#### 2.1.1. Identification of Up-/Down -Regulated Metabolites

Plasma samples from both untreated or treated (low, medium or high dose xanthohumol) male and female Zucker rats were analyzed by UPLC-MS^E^ and UPLC-IMS-MS^E^ in positive and negative mode. Initially, we used unsupervised principal component analysis (PCA) to evaluate the data sets and to obtain a first indication of general separation trends. This indicated that groups were separated by gender (Supplemental Materials, Figure S1); therefore, we evaluated female and male treatment groups separately. The PCA scores plot generated from the female rat data sets of all four groups (control and three xanthohumol treatment groups) indicated that the xanthohumol treatment groups showed little separation tendency, whereas the data sets of the male rats showed clear separation between control and the different dose groups (Supplemental Materials, Figure S2). This observation is consistent with previous findings by Legette *et al.* that showed XN treatment caused loss of bodyweight in male, but not in female, obese Zucker rats [32].

**Figure 1:**
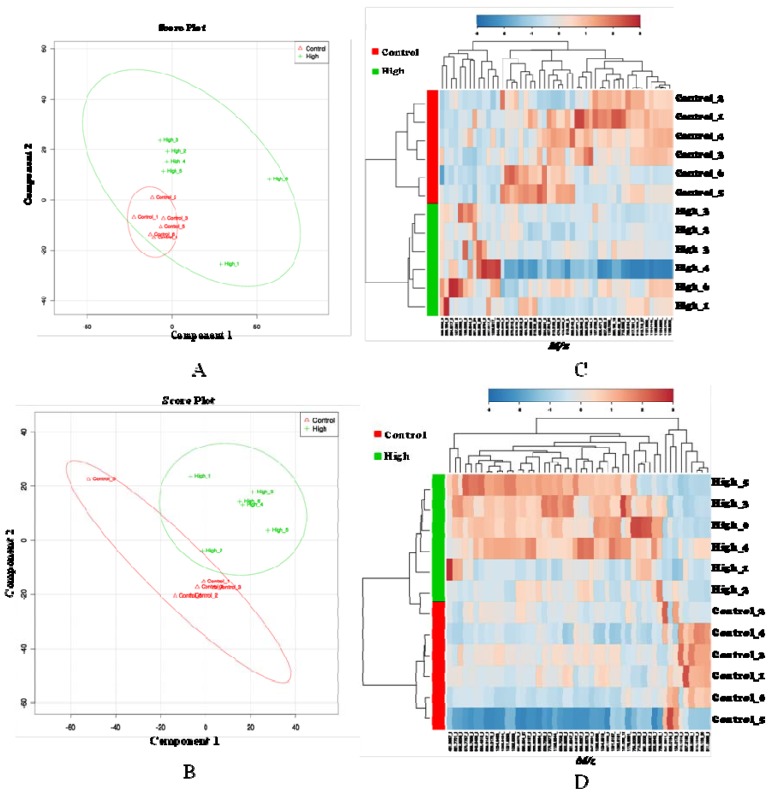
Partial Least Square-Discriminant Analysis (PLS-DA) plot (**A**, **B**) and unsupervised hierarchical clustering plot (**C**, **D**) for control and high dose groups (male animals). (A, C) positive ionization mode; (B, D) negative ionization mode; a heat map was constructed using the top 50 important metabolites created from MetaboAnalyst software (2.0). The metabolites and samples were hierarchically clustered by the Ward algorithm using Euclidian distance. Each column represents a unique feature with a characteristic mass-to-charge ratio and retention time value. As shown in the heat map alignment, the two groups were clustered by an unsupervised algorithm, which confirms the presence of discriminating features between the high dose and control groups.

We, therefore, concentrated our metabolomic analyses on the data sets obtained from the male rat plasma samples of the control group (n = 6) and the group treated with the high dose of xanthohumol (n = 6). Pre-processing of the data using XCMS, an open-source metabolomic data processing software (http://metlin.scripps.edu/xcms/), resulted in a three-dimensional matrix, mass-to-charge ratio (*m/z*), retention time (RT) and intensity values, consisting of 4,331 and 4,019 features in the positive and negative mode, respectively. These features were used for both univariate and multivariate data analysis. The scores plot showed a separation tendency between the control and the treatment group. Consequently, a Partial Least Square-Discriminant Analysis (PLS-DA) was performed to identify discriminating features between the groups. As shown in Figure 1, top left, the control group clustered well and is separated from the treatment (high dose) group; however, treated samples were spread out. Student *t*-tests were performed for statistical comparisons between the control and treated group, and *p*-values of 0.05 or less were considered significant. Further visualization of data was carried out using a heat map with two way-hierarchical clustering using the Ward algorithm and Euclidian distance on the top 50 discriminating features between the two study groups. This hierarchical clustering resulted in better separation of the control group from the treated group by reducing the variation in the treated group (Figure 1A, B). Finally, the candidate markers were selected by examining the volcano plot and considering a fold change threshold of 1.5 and a *p*-value less than 0.05 (Supplemental Materials, Figure S3). In addition, XN and XN-related metabolites [31,33] were excluded from the candidate marker list before performing the database searches. A total of 121 features were selected for accurate mass-based annotation using the Metlin, Lipidmaps, Madison Metabolomics Consortium Database (MMCD) and Human Metabolome Database (HMDB) databases. Differential metabolites are listed in the Supplemental Materials, Table 1.

**Figure 2:**
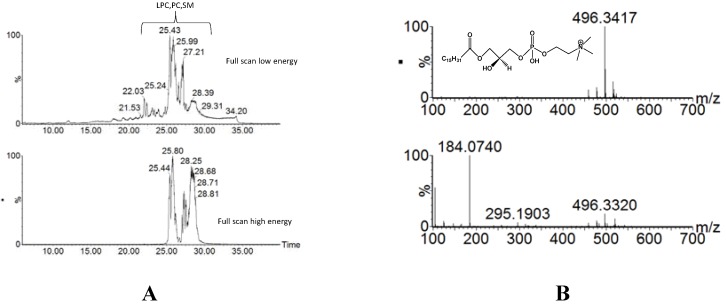
(**A**) Full scan mass spectrometry (MS) chromatograms obtained in MS^E ^ mode using low (top) and high (bottom) energy regimes. Extracted ion chromatogram (XIC) obtained with an *m/z* of 184.0740 (phosphocholine head group) enables localization of the elution window for lysophosphatidyl choline (Lyso-PC), phosphatidyl cholines (PC) and sphingomyelin (SM) lipid classes. (**B**) Extracted mass spectra of low- (top) and high- (bottom) collision energy for [Lyso-PC (16:0) + H]^+^ − (C_24_H_51_NO_7_P).The low-energy spectrum contains the precursor ion at *m*/*z* 496.3417 ([M+H]^+^), whereas the high-energy spectrum is dominated by the fragment ion for the phosphocholine head group (*m*/*z* 184.0740).

In the current study we utilized the MS^E^ mode, which acquires low energy full scan spectra and high energy fragmentation spectra in a single run. MS^E^ data provides access to exact mass information for precursor, the fragment ion and neutral loss data to search for diagnostic ions or common neutral losses. In the current study, we focused on the impact of xanthohumol treatment on lipid species. Utilization of MS^E^ allowed us to extract mass spectral data of distinct lipid classes based on class-specific fragment ions. For example, Figure 2B shows the extracted ion chromatogram (XIC) for an *m/z* of 184.07, characteristic for the phosphocholine head group, which enabled the extraction of elution profiles for the following lipid classes: lysophosphatidyl choline (Lyso-PC), phosphatidyl cholines (PC) and sphingomyelin (SM) lipids. Accurate mass measurements and high mass spectral resolution in both the low and the high energy mode allowed for higher confidence in metabolite assignments. 

Lipid identifications were carried out by the Metabosearch software with four embedded metabolite databases. There were many *m/z* values that did not match any of the database entries. This is a common phenomenon associated with untargeted metabolomic data and is at least partly attributable to possible adduct formation (Na^+^, NH_4_^+^) and/or in-source fragmentation (loss of H_2_O, loss of CO_2_). Lipids that showed significant fold changes (Student *t*-test, *p* < 0.05) are compiled in Table 1. The observed changes of different lipid classes suggest that xanthohumol affects lipid metabolism.

Based on the fold-change values obtained with the treated *vs.* control group, the level of plasma fatty acyl groups increased in the treated group. In contrast, plasma phosphatidic acid (PA) levels were lower in the treated animals compared to the controls. PAs are important intermediates in the biosynthesis of triacylglycerols and phospholipids and play an important role as signaling molecules [34]. Noteworthy, most of the phosphatidic acids were detected as Lyso-PA species. It was reported that the level and the composition of Lyso-PA changes rapidly due to external stimulants and inflammatory diseases. Lyso-PAs have been linked to metabolic defects associated with obesity [32,35]. Obesity is a chronic inflammatory disease. Under conditions of inflammation, phospholipases are activated and hydrolyze the *sn-1* (PLA_1_) or *sn-2* (PLA_2_) acyl bond of phospholipids [36,37]. The ratio of PC/Lyso-PC has been reported as an indicator for inflammation [38]. We examined the sum of the normalized response of the PC/Lyso-PC ratio for the control and high dose group, and about a 10%–20% variation for the PC/Lyso-PC ratio was observed with the high dose treatment. For example, the fold change (treated/control) for the PC/Lyso-PC ratio between PC (18:0/18:3) and Lyso-PC (18:0) was calculated to be 1.2, which suggests that high dose treatment with xanthohumol reduced inflammation.

**Table 1:** Differential lipids in control *vs.* treated groups. Metabolites with identification probability < 0.05 and a fold change (FC) threshold > 1.5, with a mass error < 5 ppm, were selected as significant features in both positive and negative ionization modes. Comparison of the distributions of differential lipids within the control and high treatment groups are provided as box-and-whisker plots in Supplementary Figure 4. Putative compound identifications were based on the accurate mass measurements and confirmed by high energy fragmentations.

Putative metabolites	Up-/down-regulation (T/C)	m/*z*	Molecular formula	Mass error (ppm)	Detected fragments
Hexadecanedioic acid	↑	285.206	C_16_H_30_O_4_	−2.45	[M–H–H_2_O]^−^
Lauroyl–EA	↓	244.228	C_14_H_29_NO_2_	2.87	[M+H–H_2_O]^+^
Octadecanedioic acid	↑	313.237	C_18_H_34_O_4_	−3.51	[M–H–H_2_O]^−^
PA(18:1/0:0)	↓	435.254	C_21_H_41_O_7_P	4.82	[FA 18:1–H]^−^
PA(18:2/0:0)	↓	433.236	C_21_H_39_O_7_P	−1.15	[FA 18:2–H]^−^
PA(20:4/0:0)	↓	457.235	C_23_H_39_O_7_P	−3.5	153 [C_3_H_6_PO_5_]^−^
PA(O–16:0/18:2)	↓	657.485	C_37_H_71_O_7_P	−1.67	[FA 18:2–H]^−^
PG(18:1/0:0)	↓	509.287	C_24_H_47_O_9_P	−2.55	(153 [C_3_H_6_PO_5_]^−^), [FA 18:1–H]^−^
PI(18:3/0:0)	↑	593.273	C_27_H_47_O_12_P	0.34	153 [C_3_H_6_PO_5_]^−^
PI(P–18:0/22:4)	↑	897.583	C_49_H_87_O_12_P	−3.57	153 [C_3_H_6_PO_5_]^−^
PS(18:4/22:6)	↓	826.464	C_46_H_70_NO_10_P	−3.62	(153 [C_3_H_6_PO_5_]^−^), [FA 22:6–H]^−^
PS(20:1/22:6)	↓	862.559	C_48_H_80_NO_10_P	−0.35	[M+H–185]^+^
Sphingosine–1–phosphate	↓	378.24	C_18_H_38_NO_5_P	−4.49	(79 [PO_3_]^−^), (97 [H_2_PO_4_]^−^)

T/C—treated *vs.* control; *m/z*—mass to charge ratio; PA—acylglycerophosphate; PG—phospho-glycerol; PI—phospho-inositol; PS—phospho-serine; EA—ethanol amine; FA—fatty acid.

Other lipid classes that appeared to be significantly dysregulated in the treated groups are glycerophospho-glycerols (PG), phosphatidyl serines (PS) and glycerophospho-inositols (PI). Taken together, the metabolome changes suggest that xanthohumol in the obese Zucker (fa/fa) rat alters the levels of circulating phospholipids. Up-/down-regulation of these lipid classes in the xanthohumol-treated group is consistent with the findings in a previous study that reported that xanthohumol alters cholesterol and bile acid metabolism, as well as lowers the plasma triglyceride levels of obese rats [39]. Dicarboxylic fatty acids are the products of fatty acid metabolism and can be found in high abundance when mitochondrial beta oxidation is dysfunctional [40]. We found significantly decreased levels of dicarboxylic acids in the plasma samples from xanthohumol-treated animals, which may support the notion that xanthohumol treatment enhances the mitochondrial beta oxidation process. These findings agree with the recently published rat plasma and cell culture studies conducted by Kirkwood *et al.* with similar XN treatments [41].

### 2.2. Traveling Wave Ion Mobility Mass Spectrometry (LC-IMS-MS^E^ Analysis)

During the last few years, there has been a steady increase in the use of ion mobility mass spectrometry (IMS-MS) for the rapid separation of metabolites in complex mixtures [42]. Technical advancement in instrument design and modern electronics has enabled continuous improvements in the resolution of the ion mobility device. The combination of ion mobility separators and high resolution mass analyzers is poised to impact and advance the field of metabolomics [12]. The inclusion of ion mobility separations to complex mixture analyses is twofold: first, ion mobility separation expands the separation space by adding an orthogonal dimension of separation to chromatographic separations; second, ion mobility spectrometry enables the extraction of collisional cross sections and, hence, provides an additional level of specificity to structure elucidations. The number of applications of IMS-MS for the advanced structure analyses of metabolites of small molecule drugs is growing and includes the elucidation of the hydroxylation and glucuronidation patterns of small molecule drugs [43], applications to untargeted metabolome analyses [44], lipid analyses [45,46] and carbohydrates [47,48,49]. In this report, we applied travelling wave ion mobility in combination with MS^E^ acquisition (IMS-MS^E^) to reduce the congestion of ion signals of lipids that were insufficiently separated under the applied LC conditions. 

**Figure 3:**
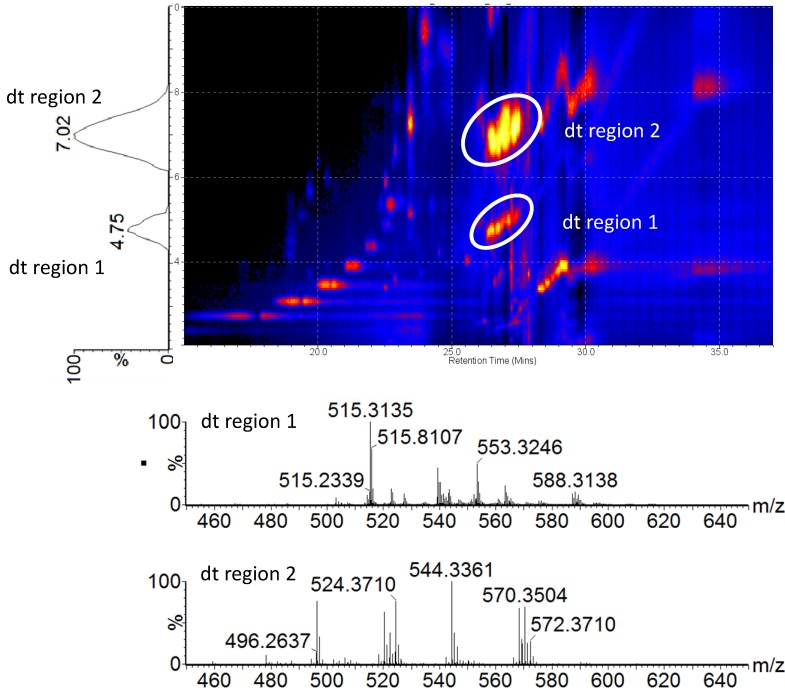
An example of a 2D image (drift time *vs.* retention time) showing the ion mobility separation of different compound classes in rat plasma samples. The encircled regions mark the compound classes that eluted within a similar retention time window (26–28 min). Drift time-extracted spectra (bottom) show that these two clusters belong to different lipid classes, namely Lyso-PC and SM lipids (sphingosine phosphocholines) that have drift time distributions centered around 7.02 ms and 4.75 ms, respectively.

**Figure 4:**
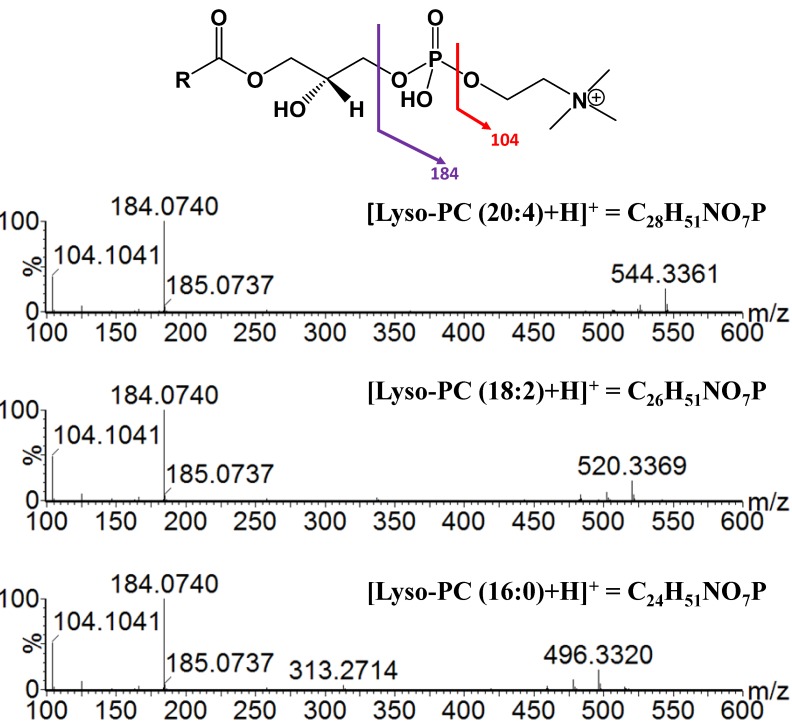
MS/MS spectra extracted from the high energy ion mobility spectrometry (IMS) function corresponding to drift time region 2 (~7.0 ms), which contains different Lyso-PCs. The general structure and the predicted fragmentation positions are given at the top. Both saturated and unsaturated ions give rise to the same characteristic fragment ion in high energy mode. The top right corner of each spectrum contains the molecular formula for the selected Lyso-PC ion.

Figure 3 shows an example of a 2D image plotted with drift time *vs.* retention time, for a plasma sample from the xanthohumol (high dose)-treated group. Ion mobility separation facilitated the detection of low abundant lipid ions by separating the lipid ions from matrix ions and other spectral interferences. As described earlier, most of the lipid classes with a polar phosphocholine head group co-eluted, because of the relatively short LC gradient used in this study. The additional separation space provided by ion mobility enabled the separation of lipid classes that co-eluted at the chromatographic scale, leading to mass spectral overlap and data crowding. As shown in Figure 3, post-acquisition spectral extraction of the two regions marked in the 2D image enabled the interrogation of the distinct lipid classes with minimal spectral interference. Based on the accurate mass measurement, the ion cluster with a drift time distribution centered on seven milliseconds belongs to Lyso-PC lipids, whereas the SM lipids show a drift time distribution around 4.75 milliseconds. PC and SM share the same characteristic fragment ion with the loss of the polar phosphocholine head group (*m/z* 184.0739). The main difference between these two lipid classes is the linkage with the phosphatidyl head group. In SM lipids, the head group is linked via an N-acyl fatty acid group with a long chain hydrocarbon [18]. In a regular MS^E^ experiment, some of the information of these co-eluting compounds can be lost due to the differences in ion abundance. Separating these compounds in an additional dimension enabled the identification of more low abundance ions and provided relatively “cleaner” spectra for both MS and MS/MS analyses. Figure 4 illustrates the high energy fragmentation of selected Lyso-PCs isolated from drift time region 2 (see Figure 3). Collision energy ramping from 15–45 eV was applied to fragment “all” ions entering into the transfer region, and the resulting spectra showed the characteristic fragmentation pattern with the loss of the phosphatidyl choline head group (*m/z* 184.0740) as the dominating ion in all the samples. With the application of high energy, the phosphatidyl head group fragmented further to give another characteristic ion with *m/z* 104.1041 ([C_5_H_14_NO]^+^). Fragmenting after IMS generates product ions that are drift time- and retention time-aligned, with their precursors providing more confident metabolite identifications with low or only minimal matrix interferences.

#### Ion Mobility-Mass Correlation Analyses

**Figure 5:**
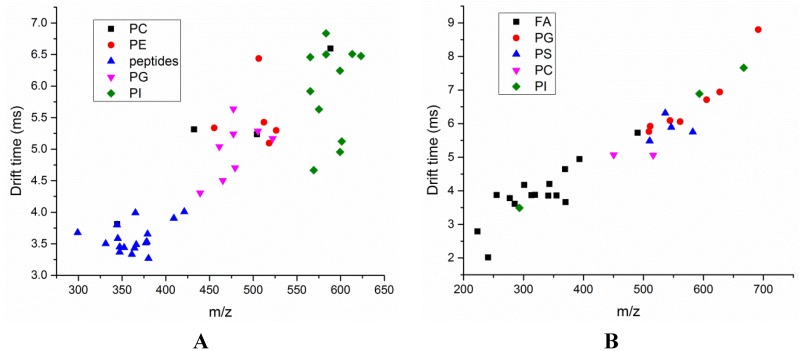
Ion mobility correlation analyses for the significant features identified in (**A**) positive and (**B**) negative ionization modes. Data were acquired in low energy IMS-MS^E^ mode, and the tentative compound identifications were obtained based on their accurate mass measurements.

After identifying the masses, ion mobility correlations were analyzed for various metabolite classes identified in the Zucker rat (fa/fa) plasma samples. Figure 5 shows the correlation plots with *m/z* on the x-axis and drift time on the y-axis. A total of five compound classes were assigned for significant metabolites detected in the negative and positive ionization modes. These correlation analyses can be used as a predictive tool to determine the compound class for an unknown metabolite. If a compound class database can be created (varying carbon chain lengths, number of double bonds) using a set of standards, these correlation analyses can be used as an additional tool for the verification of a metabolite with regard to using the *m/z* value alone. One of the caveats of this method is the occurrence of multiple charge state molecules. Multiply charged ions overlap with one another in the three-dimensional conformational space and decrease overall peak capacity. This will result in one analyte being represented by several ion signals in the spectrum. Additionally, different adduct formations can lead to false identification of compound classes with the same *m/z-*drift time pairs. 

PC—phosphatidyl cholines; PG—phospho-glycerol; PI—phospho-inositol; PS—phospho-serine; EA—ethanol amine; FA—fatty acid

## 3. Experimental Section

### 3.1. Sample Preparation

Samples were prepared according to the method described by Kirkwood *et al*. [41]. Briefly, four-week old male and female Zucker fa/fa rats were purchased from Harlan (Livermore, CA, USA). All rats were maintained on a high fat (60% kcal) AIN-93G diet for 3 weeks to induce severe obesity, followed by a normal AIN- 93G (15% kcal fat) diet for the last 3 weeks of the study. After a 2-day acclimation period following transportation, 48 animals (24 males and 24 females) were divided into four treatment groups (n = 6/gender group): control (0 mg xanthohumol /kg body weight (BW)), low (1.86 mg xanthohumol /kg BW), medium (5.64 mg xanthohumol /kg BW) and high (16.9 mg xanthohumol /kg BW). Animals were euthanized with an overdose of CO_2_. Plasma metabolites were extracted using an ice-cold methanol: ethanol mixture (sample/solvent = 1/4). Sample extracts were kept in a −80 °C freezer prior to LC-MS/MS analysis. A quality control (QC) sample was prepared by pooling 10 µL from each of the biological samples.

### 3.2. LC-MS Analysis

#### 3.2.1. LC Setup

High-performance liquid chromatography was performed on a Shimadzu Nexera system (Shimadzu, Columbia, MD, USA) coupled to a Synapt G2 HDMS mass spectrometer (Waters, Manchester, UK). Chromatographic separations were carried out on an Inertsil phenyl-3 column (150 × 4.6 mm, 5 µm, MetaChem Technologies, Torrance, CA, USA) for positive ion and negative ion analysis. Flow rate was set to 0.4 mL/min, and the mobile phases consisted of water (A) and methanol (B), both with 0.1% formic acid. The elution gradient was as follows: 0 min, 5% B; 1 min, 5% B; 11 min, 30% B; 23 min, 100% B; 35 min, 100% B; 37 min, 5% B; and 47 min, 5% B. Column temperature was held at 70 °C, and the injection volume was 10 µL.

#### 3.2.2. MS^E^

The inlet (HPLC system) was coupled to a hybrid quadrupole orthogonal time-of-flight mass spectrometer (SYNAPT-G2 HDMS, Waters, MS Technologies, Manchester, UK). Electrospray positive and negative ionization modes were used for the analysis. A capillary voltage and cone voltage of −3 kV and −35 V, respectively, were used in both polarities. The desolvation source conditions were as follows: for the desolvation gas, 550 L/h was used, and the desolvation temperature was kept at 400 °C. Data acquisition took place over the mass range of 50–1200 Da in centroid MS^E^ mode. The reference internal calibrant (Leucine enkephalin, *m/z* 556.2771 for positive ion mode and *m/z* 554.2615 for negative ion mode) was introduced into the lock mass sprayer at a constant flow rate of 10 µL/min. Lock mass acquisition was controlled automatically by the acquisition software, and a reference scan was conducted every 10 s lasting 0.3 s. The mass spectrometer was calibrated with sodium formate in both positive and negative ionization modes. During this acquisition method, the first quadrupole, Q1, is operated in a wide band radio frequency (RF) mode only, allowing all ions to enter the T-wave collision cell. Two discrete and independent interleaved acquisition functions are automatically created. The first function, typically set at 5 eV, collects low energy or unfragmented data, while the second function collects high energy or fragmented data typically set by using a collision energy ramp from 15–45 eV. In both instances, Argon gas was used for collision-induced dissociation.

#### 3.2.3. IMS-MS^E^

Similar ionization parameters were used to acquire continuum data in IMS-MS^E^ mode. The trap, IMS and transfer traveling wave devices were operated with traveling wave amplitudes and velocities of 8.0 V and 508 m/s, 40.0 V and 1,000 m/s and 3.0 V and 450 m/s, respectively. The trap/transfer cells were operated with argon at a pressure of 2.5 × 10^−2^ mbar. Nitrogen as the drift gas was introduced into the IMS cell to maintain a pressure of 3.1 mbar. The ToF analyzer was operated in the V-mode with an average mass resolution of m/Δm 20,000 (full-width at half-maximum definition). The ToF pusher and trap-gate frequencies were 18.5 kHz and 92.5 Hz, respectively. Data acquisition and processing were performed using MassLynx (V4.1, SCN714), Driftscope (V2.2) and MS^E^ viewer (V1.2).

### 3.3. LC-MS Data Preprocessing and Statistical Analysis

MS^E^ data were converted into NetCDF format (Network Common Data Form) using MassLynx software (Waters Corp, Milford, MA). Converted data sets were analyzed by the XCMS program using the R package (2.15.1) for initial data processing, including peak grouping, retention time alignment and filling in missing features [50]. The resulting data matrices were then imported into MetaboAnalyst software (2.0) for statistical analysis [51,52]. Xanthohumol and its related metabolites were excluded from the sample list prior to statistical validation [31,33]. Features in a data matrix were filtered based on relative standard deviation (RSD) of whole intensity values, and log transformation and auto-scaling were incorporated to ensure equal contributions from each variable to the models. Partial Least Square Discriminant Analysis (PLS-DA) was performed to identify discriminating features between control treatment groups. Data visualization using unsupervised, two-way hierarchical clustering was performed using the Ward algorithm using the Euclidian distance on the normalized metabolites. A Student *t*-test was performed for statistical comparison between control and high dose rat samples, and a *p*-value of 0.05 or less was considered significant. Significant features were selected based on the *p*-value (*p* < 0.05) and fold change threshold (FC > 1.5). MetaboSearch was used to perform *m/z*-based searches simultaneously against the four major metabolite databases: Human Metabolome Database (HMDB), Madison Metabolomics Consortium Database (MMCD), Metlin and LipidMaps [53]. Finally, the compound assignments were confirmed by matching the high energy MS^E^ data with the reported and predicted fragmentation patterns for selected metabolites.

## 4. Conclusions

Application of MS^E^ and IMS-MS^E^ for untargeted metabolomics has been demonstrated for plasma samples obtained from obese Zucker (fa/fa) rats that were treated with different oral doses of xanthohumol. Data-independent MS^E^ allowed the acquisition of both low energy and high energy spectra in the same analysis. Addition of ion mobility as an additional separation dimension enabled the detection and identification of low abundant sphingolipids in a congested mass spectral region. Based on the dose response studies, the differential metabolites identified confirmed the anti-inflammatory properties of xanthohumol and further suggest that xanthohumol affects lipid metabolism in obese Zucker (fa/fa) rats. 

## Supplementary Files

Supplementary File 1 Supplementary (PDF, 339 KB)
