# Supplementary material for: Electrospray Quadrupole Travelling Wave Ion Mobility Time-of-Flight Mass Spectrometry for the Detection of Plasma Metabolome Changes Caused by Xanthohumol in Obese Zucker (fa/fa) Rats
Source: Metabolites. 2013 Aug 13;3(3):701–17. doi: 10.3390/metabo3030701 (PMC3901285; doi:10.3390/metabo3030701)
Supplement: Supplementary File 1 — Supplementary (PDF, 339 KB) [file metabolites-03-00701-s001.pdf]

Supplementary Materials

Figure S1. Partial Least Square Discriminant Anslysis (PLS-DA) scores plot for male (+) and female (Δ) plasma sample with XN treatments. Elipse shows the 95% confidence region.

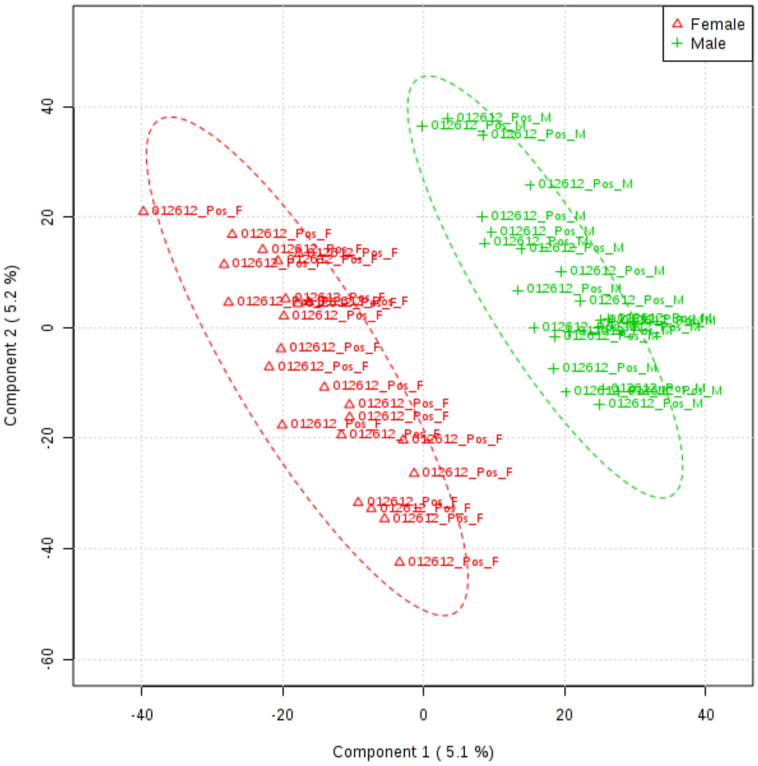

Figure S2. PLS-DA scores plot for control (Δ) and different dose groups low- x; medium-◊; high-+ (plasma, <u>male)</u>.Eclipse shows the 95% confidence region.

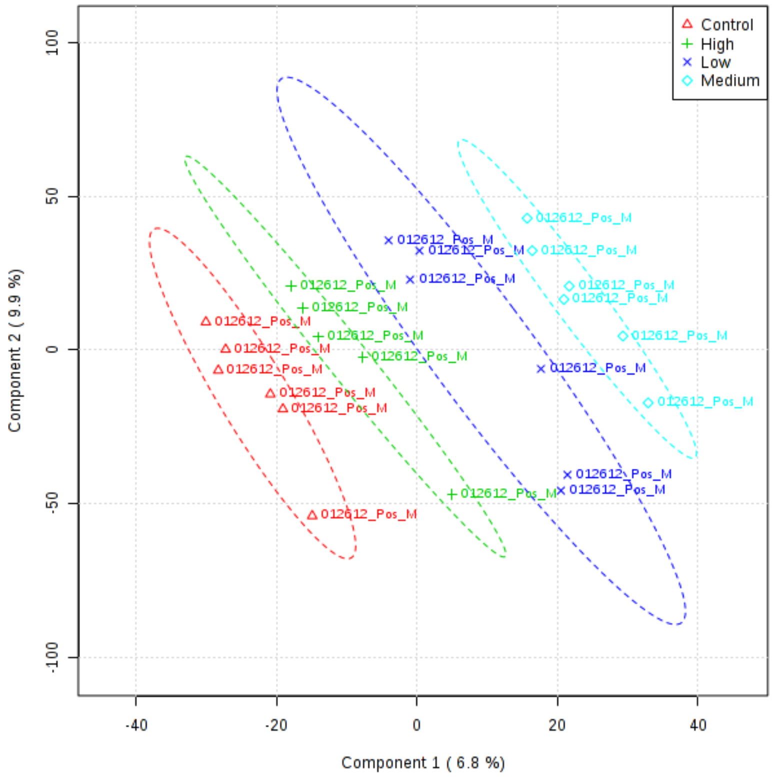

Figure S3. Volcano plots generated for control *vs*. high dose group (plasma, <u>male</u>) in negative ionization mode (left) and positive ionization mode (right). Each point represents a feature plotted as a function of fold-change (Log2 (fold change), *x*-axis) and statistical significance (−Log10 (*p*-value), *y*-axis). Vertical dotted lines represent fold changes of ±1.5. The horizontal dotted line corresponds to the minimum *p*-value of selected metabolites (*p*-value < 0.05). The pink dots represent selected putative markers *p*-value < 0.05 and fold change < 1.5.

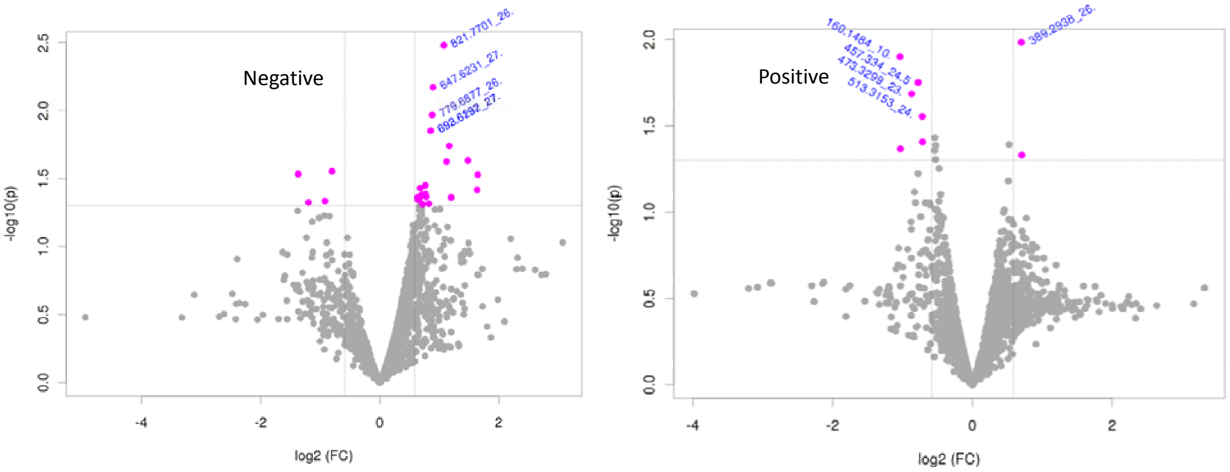

Table S1. Plasma differential metabolites obtained from Zucker (fa/fa) rats with (high dose) and without (control) xanthohumol treatment.

**Metabolites**	**Mode**	**m/*z***	**Molecular Formula**	**RT**	**Fold Change (T/C)**	***p*-Value**	**Class**
3-Methoxy-4-hydroxyphenylglycolaldehyde	Negative	181.0520	$C_9H_{10}O_4$	12.40	3.3346	0.0349	Tyrosine metabolism
5-Hydroxyindoleacetate	Negative	190.0500	$C_{10}H_9NO_3$	12.01	0.6377	0.0250	Tryptophan metabolism
Guanidinoethyl methyl phosphate	Negative	196.0479	$C_4H_{12}N_3O_4P$	19.96	0.4838	0.0224	Enzymatic rxn
Hexadecanedioic acid	Negative	285.2064	$C_{16}H_{30}O_4$	24.66	1.5300	0.0392	Fatty Acyls [FA]
dUMP	Negative	307.0341	$C_9H_{13}N_2O_8P$	5.89	1.1287	0.0368	Pyrimidine metabolism
Octadecanedioic acid	Negative	313.2373	$C_{18}H_{34}O_4$	25.22	1.6276	0.0262	Fatty Acyls [FA]
4-O-beta-D-Glucosyl-4-hydroxycinnamate	Negative	325.0953	$C_{15}H_{18}O_8$	18.46	0.3965	0.0497	Enzymatic rxn in UDP-glucose
2;3-dinor Thromboxane B1	Negative	343.2117	$C_{18}H_{32}O_6$	24.56	1.3326	0.0302	major metabolite of thromboxane B2
Sphingosine-1-phosphate	Negative	378.2398	$C_{18}H_{38}NO_5P$	24.27	1.2891	0.0477	Sphingolipids [SP]
PA(18:2/0:0)	Negative	433.2356	$C_{21}H_{39}O_7P$	25.31	0.6159	0.0276	Glycerophospholipids [GP]
PA(18:1/0:0)	Negative	435.2538	$C_{21}H_{41}O_7P$	25.07	1.5474	0.0498	Glycerophospholipids [GP]
PA(20:4/0:0)	Negative	457.2345	$C_{23}H_{39}O_7P$	27.14	1.1679	0.0133	Glycerophospholipids [GP]
PG(18:1/0:0)	Negative	509.2872	$C_{24}H_{47}O_9P$	24.61	1.2307	0.0500	Glycerophospholipids [GP]
24-methylcholest-22-en-3,4,5,6,8,14,15,25,28-nonol	Negative	527.3246	$C_{28}H_{48}O_9$	25.63	0.6111	0.0167	Sterol Lipids [ST]
PI(18:3/0:0)	Negative	593.2734	$C_{27}H_{47}O_{12}P$	24.81	1.8090	0.0357	Glycerophospholipids [GP]
PA(O-16:0/18:3)	Negative	655.4662	$C_{37}H_{69}O_7P$	27.18	1.4419	0.0405	Glycerophospholipids [GP]
PA(O-16:0/18:2)	Negative	657.4854	$C_{37}H_{71}O_7P$	27.12	1.2782	0.0255	Glycerophospholipids [GP]
Anthenoside A	Negative	678.4571	$C_{38}H_{65}NO_9$	26.38	0.6100	0.0302	Sterol Lipids [ST]
PS(18:4/22:6)	Negative	826.4635	$C_{46}H_{70}NO_{10}P$	26.70	0.8954	0.0489	Glycerophospholipids [GP]
PI(P-18:0/22:4)	Negative	897.5830	$C_{49}H_{87}O_{12}P$	24.80	1.6147	0.0472	Glycerophospholipids [GP]

Table S1. *Cont.*

Metabolites	Mode	m/z	Molecular Formula	RT	Fold Change (T/C)	p-Value	Class
11-amino-undecanoic acid	Positive	202.1817	$C_{11}H_{23}NO_2$	27.09	0.8197	0.0480	Fatty Acyls [FA]
2-hydroxy-tridecanoic acid	Positive	231.1952	$C_{13}H_{26}O_3$	26.98	0.8538	0.0329	Fatty Acids
Lauroyl-EA	Positive	244.2278	$C_{14}H_{29}NO_2$	26.99	0.8831	0.0411	Fatty Acyls [FA]
Uridine	Positive	245.0775	$C_9H_{12}N_2O_6$	13.55	1.0666	0.0216	Pyrimidine metabolism
N-(L-Arginino)succinate	Positive	291.1280	$C_{10}H_{18}N_4O_6$	24.51	0.7727	0.0161	Alanine, aspartate and glutamate metabolism
(22)-1,22,25-trihydroxy-26,27-dimethyl-23,23,24,24-tetradehydrovitamin D3	Positive	457.3340	$C_{29}H_{44}O_4$	24.57	1.5379	0.0389	Sterol Lipids [ST]
3-dehydroecdysone	Positive	463.3062	$C_{27}H_{42}O_6$	26.35	0.6908	0.0224	Cholesterol
Cucurbitacin H	Positive	535.3276	$C_{30}H_{46}O_8$	26.38	0.8705	0.0419	Sterol Lipids [ST]
PC(18:0/20:4(12OH[S]))	Positive	826.6013	$C_{46}H_{84}NO_9P$	26.36	0.6701	0.0154	Glycerophospholipids [GP]
PE(22:4/22:6)	Positive	840.5483	$C_{49}H_{78}NO_8P$	25.57	0.6860	0.0443	Glycerophospholipids [GP]
PS(20:1/22:6)	Positive	862.5590	$C_{48}H_{80}NO_{10}P$	25.57	0.6783	0.0364	Glycerophospholipids [GP]
TG(20:2/20:3/20:3)	Positive	959.7993	$C_{63}H_{106}O_6$	22.39	0.8216	0.0375	Glycerolipids [GL]
PE(26:2/26:2)	Positive	964.7657	$C_{57}H_{106}NO_8P$	25.31	0.7242	0.0459	Glycerophospholipids [GP]

T/C- Treated vs Control, *m/z*- mass to charge ratio, PA-acylglycero phosphate,PC- phospho choline, PE-phospho ethonolamine, PG-phospho glycerol, PI-phospho inositol, PS-phospho serine, EA-ethanol amine, FA-fatty acid
